# Half of microbial eukaryote literature focuses on only 12 human parasites

**DOI:** 10.1093/ismejo/wraf219

**Published:** 2025-10-07

**Authors:** Joanna A Lepper, H Beryl Rappaport, Angela M Oliverio

**Affiliations:** Department of Biology, Syracuse University, Syracuse, NY 13210, United States; Department of Biology, Syracuse University, Syracuse, NY 13210, United States; Department of Biology, Syracuse University, Syracuse, NY 13210, United States

**Keywords:** microbial eukaryotes, protists, eukaryotic tree of life, biodiversity

## Abstract

Although microbial eukaryotes comprise the majority of eukaryotic phylogenetic diversity and inhabit nearly all ecosystems globally, most research focuses on only a few species of human parasites. Here, we quantify the extent of research on known microbial eukaryotic species. Nearly half of the mentions of protist species on publications in PubMed referenced only 10 species included in the Protist Ribosomal Reference (PR2) Database. Likewise, although most samples in the PR2 database are free-living protists from aquatic environments, 12 species of human parasites comprise 47% of the literature. Research efforts that focus on only a handful of eukaryotic lineages severely limit our understanding of the fundamental biology of eukaryotic cells. We highlight recent efforts to characterize novel eukaryotic lineages that have resulted in a new understanding of the rules of life and identify key lineages that are notably absent or limited in the literature, despite their abundance and significance across global ecosystems.

Although microbial eukaryotes (e.g. protists) comprise the vast majority of eukaryotic life on Earth [1], most research efforts focus on only a handful of lineages, limiting our understanding of eukaryotic biology. Characterizing non-model eukaryotes has repeatedly broadened our view on what is possible in biology [2]. For example, it was previously thought that nitrogen fixation was exclusive to prokaryotic cells, but recent research on a species of algae, *Braarudosphaera bigelowii,* led to the discovery of a new organelle in eukaryotic cells, the nitroplast [3]. Likewise, research in ciliates [4] led to the discovery of non-canonical genetic codes.

In an effort to shine light on the bias within protist research, we performed a quantitative assessment of the number of published articles across microbial eukaryotic species. Inspired by a recent parallel effort in bacteria [5], we quantified the extent of research bias in microbial eukaryotes. We first performed a bibliometric analysis of the PubMed database, counting the number of publications for each species of protist (genus and species name in the title or abstract). We used the Protist Ribosomal Reference (PR2) Database to obtain a list of named protist species, resulting in 8456 species mentioned a total of 242 844 times. We emphasize that our analyses are limited to species with named taxonomies. The actual number of protist species is much greater, with estimates ranging from 2 to 10 million or more species [6]. This underscores the extreme lack of characterization across the eukaryotic tree of life. More protist species are denoted in NCBI than PR2 (~60 000 species [7]), but many lack species names and/or corresponding sequence data, restricting our ability to match NCBI species to the PubMed database. Although named species comprise only a small proportion of eukaryotic diversity (Fig. 1A), these data provide a valuable window into current biases in research efforts.

**Figure 1:**
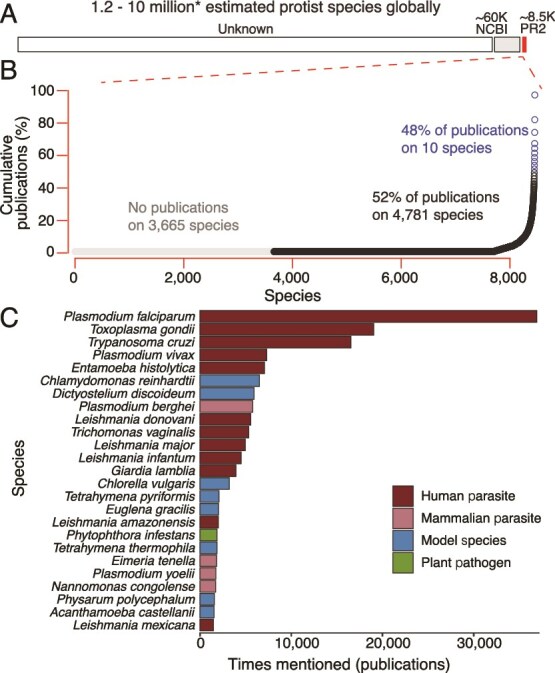
The vast majority of microbial eukaryotic research is focused on a small number of species, mostly human and mammalian parasites. (A) A bar plot showing the estimated number of protist species globally versus those that have been described in NCBI and PR2 databases. There are an estimated 1.2–10 million species of protists. Of that, NCBI acknowledges about 60,000 [7]. The PR2 database, used in this study, has ~8,500 unique species of protists. (B) The top 10 protist species make up 48% of the publications in PubMed, and 3,665 species remain unstudied. Blue circles indicate the top ten species, black circles indicate other species detected in the PubMed literature, and gray circles indicate those species that had no title or abstract matches in PubMed. (C) A bar plot highlighting the skewed distribution of publications on protists, with the top 25 making up 62.7% of all protist publications. Each bar represents the number of publications in which each protist is mentioned. The most studied type is human parasites.

We found that the top 10 species comprise nearly half (48%) of the literature on microbial eukaryotes. Further, the top 25 protist species make up nearly two-thirds of all published research (63%; Fig. 1B, Table S1). The most frequently mentioned species are primarily human parasites. Twelve parasites (including *Plasmodium falciparum*, *Toxoplasma gondii*, *Trypanosoma cruzi*, and *Plasmodium vivax*) collectively make up 75% of the top 25 species and 47% of all PubMed literature on protists. *Plasmodium falciparum*, the parasite responsible for malaria in humans, was the most mentioned protist, in 39 915 publications and comprising 15% of the total literature. In addition, 43% of the species in PR2 were not mentioned in a single research article on PubMed.

Beyond parasites, the top 25 species also included eight lineages considered nonparasitic model species for protists, such as *Chlamydomonas reinhardtii* and *Dictyostelium discoideum* (Fig. 1C). Even most microbial eukaryotic model species are far less developed than *Escherichia coli* or *Saccharomyces cerevisiae*, with limited or no genetic tools available [8]. Although both *E. coli* and the protist *C. reinhardtii* were developed as model organisms in the 1940s [9, 10], the difference in accumulated knowledge between them is drastic. *E. coli* has over 300 000 hits on PubMed, while *C. reinhardtii* has only 6504 hits and lags far behind in genetic tool development and characterization of its basic biology and interactions with other microbes. Incredibly, there are more mentions of *E. coli* alone than all protists. Our understanding of even the best studied microbial eukaryotes lags behind that of other microbial groups.

We observed a large discrepancy between the sampling sources of the most frequently sequenced protists versus those most frequently discussed in the literature (Fig. 2). Although research efforts on protists are dominated by mammalian parasites found in blood (Fig. 2A), the most frequently sequenced protists were from aquatic habitats including both marine and freshwater environments (Fig. 2B). Sequenced species were mostly free-living, often planktonic, and included several clades of poorly described dinoflagellates, diatoms (*Bacillariophyceae* and *Mediophyceae*), and ciliates (*Sessilida*, *Oligotrichida*, and *Strombidiidae*). The habitats from which protists are most often collected and sequenced does not translate to those most studied in the literature. It is important to note that the most sequenced protists also reflect a bias in sampling of aquatic environments relative to other habitats including terrestrial soils, in which protists are known to be abundant and diverse. None of the top 30 protists were sampled from soil, although soil hosts orders of magnitude more species diversity than aquatic environments [11, 12].

**Figure 2:**
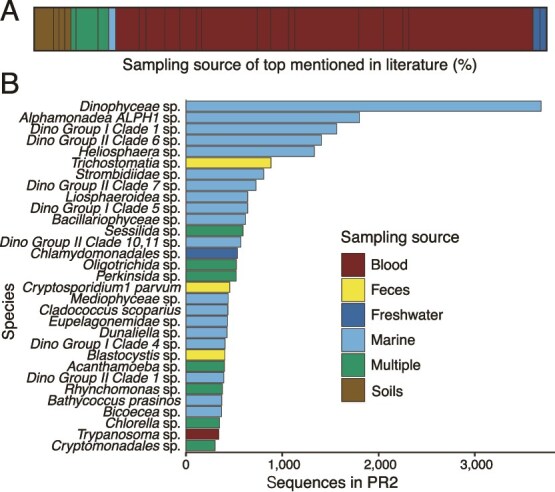
There is a mismatch between the sampling source of protists most mentioned in literature versus the sources where protists are most frequently sampled. (A) A bar plot summarizing the top mentioned species on PubMed. Black bars represent distinct species and are scaled by relative percent of mentions. Colors represent the reported sampling source (including blood, feces, freshwater, marine, soils, and multiple sources reported). (B) A bar plot displaying the top sequenced species in the PR2 database. The most frequently sampled species are primarily sourced from marine environments, and nearly all are aquatic, with only a few found in feces or blood. The multiple category is composed of species sampled from multiple environments.

To contextualize research effort of protists in a phylogenetic framework, we built a tree of unique protist sequences annotated with the corresponding number of mentions in PubMed. Several major clades including stramenopiles, dinoflagellates, and rhizarians had a large number of distinct sequences but no representatives in the top 20 most mentioned protists. The stramenopiles exhibit a remarkable ecological and functional diversity, from oxygen-producing algae and kelp to pathogenic oomycetes. Intriguingly, they also represent the group with the highest proportion of unstudied species (Fig. S1). Another major clade for which we detected a large number of environmental sequences but that remains poorly studied is *Rhizaria*. Recent work in *Foraminifera* challenges the long-held paradigm that genomes predominately cycle between haploid and diploid states [13]. *Allogromia laticollaris* individuals spend most of their time in an endoreplicated stage [13]. Additional characterization of species within these clades is likely to yield further insight on eukaryotic cell biology.

Even the best studied lineages are nested within clades of mostly unknown species. *Apicomplexa* lineages (within *Alveolata*) comprise a majority of the top studied species, (e.g. *Plasmodium*, *Toxoplasma*; Fig. S2A). Yet most *Apicomplexa* have only been detected *via* environmental sequencing [14], and other major clades within *Alveolata*, including dinoflagellates and ciliates, are far less referenced (Fig. S2B). Dinoflagellates may be of interest due to their unique genome structure and gene regulation [15]. Ciliates have already challenged the rules of biology with widespread non-canonical genetic codes and unusual genomic architecture [16, 17]. Increased characterization efforts leveraging cutting-edge methods (such as RNA interference tools, high throughput proteomics, and cryo-electron microscopy) can help their progress in becoming tractable models [18].

We also quantified the relative research effort of fungal species compared to all microbial eukaryotes (Fig. S3). Although fungi represent only a small fraction of eukaryotic phylogenetic diversity, *S. cerevisiae* and *Candida albicans* (Fig. S3) both have a greater number of mentions than any protist. *S. cerevisiae* has 38 227 more mentions than *P. falciparum*, the most mentioned protist. Fungi were mentioned 320 031 times versus 242 844 for all microbial eukaryotes, underscoring the unbalanced research emphasis.

Recognized for their human health consequences, parasites have overwhelmingly received the most research among protists, but they are a small minority of protist species: most are free-living. Parasites are also poorly representative of eukaryotic diversity, often with reduced genomes and fast evolutionary rates [19, 20]. Description of unknown lineages across understudied ecosystems is a key source of discovery for lineages that do biology differently.

## Supplementary Material

Supplementary_Information_ISME_Protist_Lit_wraf219

## Data Availability

All code and data used in the analyses are available at: https://doi.org/10.6084/m9.figshare.c.7862267.
