# Supplementary material for: Half of microbial eukaryote literature focuses on only 12 human parasites
Source: ISME J. 2025 Oct 7;19(1):wraf219. doi: 10.1093/ismejo/wraf219 (PMC12560768; doi:10.1093/ismejo/wraf219)
Supplement: Supplementary_Information_ISME_Protist_Lit_wraf219 [file supplementary_information_isme_protist_lit_wraf219.docx]

**Supplementary Information**

**Supplementary Methods:**

To compile a list of protists from the PR2 Database, plants, animals, and fungi were filtered out, as well as any samples that did not have an 18S rRNA sequence. Separately, fungi were added back in to compare the research effort of fungi relative to protists. To first look at bias in literature, the number of publications for each species were found by using the PubMedR (in PubMed as of April 11, 2025) package in Rstudio. Full names (e.g. *Plasmodium falciparum*) were searched for in titles and abstracts to find the number of publications associated with a species, as some species were common phrases (e.g. ammonia) and some abbreviated names (e.g. *A. acuta*) were duplicated. We relied on current taxonomic names as assigned in PR2 and did not make additional manual modifications to merge, split, or revise species names. For example, PR2 uses *Giardia lamblia* so this is preserved, although we note the current preferred scientific name is *Giardia duodenalis*. Analyses were completed in R via ggplot and base R packages. Lists of the unique species with each corresponding number of publications for protists and fungi are on figshare. In order to highlight sampling bias, locations of samples from the PR2 Database were referenced. The location descriptions were not normalized, and not every sample had a listed source. A list of locations provided by the PR2 database is on figshare.

To look at phylogenetic bias, a single representative entry was chosen for each species and species name, division, and 18S rRNA sequences were exported from the PR2 database. This resulted in 8,470 sequences (and one outgroup sequence; Promethearchaeota archaeon Accession # MW959035), which were run through mafft alignment with default parameters, trimmed via the bioconda package trimAl[[1]](https://www.zotero.org/google-docs/?TEKx2W), and then used to build a phylogenetic tree via RaxML-HPC BlackBox algorithm on CIPRES Science Gateway[[2, 3]](https://www.zotero.org/google-docs/?vay8HN), and visualized on iTOL (Interactive Tree of Life[[4]](https://www.zotero.org/google-docs/?6S55E3). Figures were finalized using Adobe Illustrator to alter color, spacing, and text labels. A cartoon tree (modified from ref. [[5, 6]](https://www.zotero.org/google-docs/?9d7oCI)), was modeled to offer context in the diversity of protists and their evolutionary proximity to fungi.

**Supplementary References**

[1. Capella-Gutiérrez S, Silla-Martínez JM, Gabaldón T. trimAl: A tool for automated alignment trimming in large-scale phylogenetic analyses. *Bioinformatics* 2009;**25**:1972–1973. https://doi.org/10.1093/BIOINFORMATICS/BTP348](https://www.zotero.org/google-docs/?broken=PfnMxR)

[2. Miller MA, Pfeiffer W, Schwartz T. Creating the CIPRES Science Gateway for inference of large phylogenetic trees 2010; Gateway Computing Environments Workshop, GCE 2010 2010. https://doi.org/10.1109/GCE.2010.5676129](https://www.zotero.org/google-docs/?broken=bE6u8N)

[3. Stamatakis A. RAxML version 8: a tool for phylogenetic analysis and post-analysis of large phylogenies. *Bioinformatics* 2014;**30**:1312–1313. https://doi.org/10.1093/BIOINFORMATICS/BTU033](https://www.zotero.org/google-docs/?broken=PL6kgO)

[4. Letunic I, Bork P. Interactive Tree Of Life (iTOL): An online tool for phylogenetic tree display and annotation. *Bioinformatics* 2007;**23**:127–128. https://doi.org/10.1093/BIOINFORMATICS/BTL529](https://www.zotero.org/google-docs/?broken=XP72YR)

[5. Jamy M *et al.* Global patterns and rates of habitat transitions across the eukaryotic tree of life. *Nat Ecol Evol* 2022; **6**:1458–1470. https://doi.org/10.1038/s41559-022-01838-4](https://www.zotero.org/google-docs/?broken=UAibjI)

[6. Burki F *et al.* The new tree of eukaryotes. *TREE* 2020;**35**:43–55.](https://www.zotero.org/google-docs/?broken=rcjRDg) https://doi.org/10.1016/j.tree.2019.08.008

**Figure S1. Clades with the greatest number of named species that were not mentioned in the title or abstract of any PubMed article.** Bars represent the number of unique species in each major eukaryotic division from the PR2 database that were not detected from any article title or abstract on PubMed.


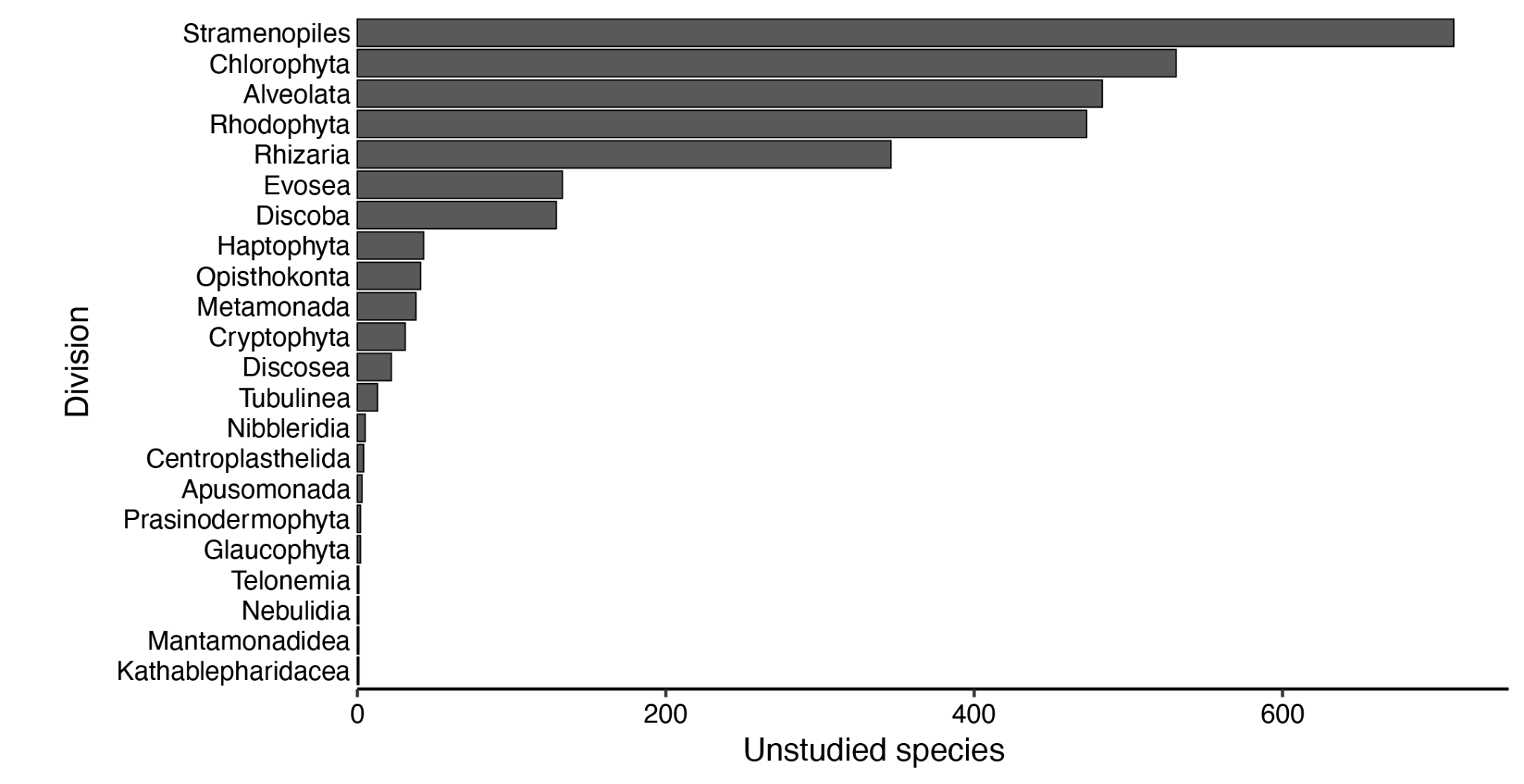


**Figure S2. Major clades of microbial eukaryotes have not been the focus of a scientific article, including most parasite diversity.** A) Phylogenetic tree of 8,470 18S rRNA sequences from protists in PR2. Sequences were aligned with mafft and the tree was built with RAxML-HPC BlackBox on CIPRES. The tree was visualized on iToL, with branches colored by major clades. Length of black bars extending from each tip is proportional to the number of times each organism is mentioned in PubMed. Some clades are unresolved due to short sequences in PR2. B) Looking into the Alveolata division reveals specific bias. Red dots represent the number of sequences in the PR2 Database for each subdivision, and black dots represent the number of publications the species of each subdivision were mentioned in.

**Figure S3. Fungi displace the top protist, but protists keep the majority of the top 25 places.** A) Cartoon tree of Eukaryotes. Fungi is highlighted in the same blue as Part B to act as a reference for the difference in diversity. Modified from ref. 4, adapted from refs. [[5, 6]](https://www.zotero.org/google-docs/?MF2UtM). B) Bar plots reflecting the top 25 microbial eukaryotes mentioned in literature when fungi are included in counts. Plants and animals are still excluded from this assessment. The PR2 database was also used for searching fungal species.

**Table S1.** **The vast majority of microbial eukaryotic research is focused on human and mammalian parasites.** Table of the top 15 protists, sorted in descending order by number of articles mentioned on PubMed.

| **Species** | **Publications** | **Percentage** | **Category** |
| --- | --- | --- | --- |
| *Plasmodium falciparum* | 39,915 | 15.20 | Human parasite |
| *Toxoplasma gondii* | 19,037 | 7.84 | Human parasite |
| *Trypanosoma cruzi* | 16,531 | 6.81 | Human parasite |
| *Plasmodium vivax* | 7,322 | 3.02 | Human parasite |
| *Entamoeba histolytica* | 7,058 | 2.91 | Human parasite |
| *Chlamydomonas reinhardtii* | 6,504 | 2.68 | Model species |
| *Dictyostelium discoideum* | 5,929 | 2.44 | Model species |
| *Plasmodium berghei* | 5,778 | 2.38 | Mammalian parasite |
| *Leishmania donovani* | 5,562 | 2.29 | Human parasite |
| *Trichomonas vaginalis* | 5,347 | 2.20 | Human parasite |
| *Leishmania major* | 4,951 | 2.04 | Human parasite |
| *Leishmania infantum* | 4,504 | 1.85 | Human parasite |
| *Giardia lamblia* | 3,932 | 1.62 | Human parasite |
| *Chlorella vulgaris* | 3,205 | 1.32 | Model species |
| *Tetrahymena pyriformis* | 2,076 | 0.85 | Model species |
